# Miscellany

**Published:** 1909-01

**Authors:** 


### MISCELLANY.

A board of commissioned medical officers will be convened to meet at the Bureau of Public Health and Marine Hospital Service, 3 B street SE., Washington, D. C., Monday, January 11, 1909, at 10 o'clock a. m., for the purpose of examining candidates for admission to the grade of assistant surgeon in the Public

Health and Marine Hospital Service. Candidates must be between 22 and 30 years of age, graduates of a reputable medical college, and must furnish testimonials from responsible persons as to their professional and moral character.

The following is the usual order of the examinations: 1, physical; 2, oral; 3, written; 4, clinical. In addition to the physical examination, candidates are required to certify that they believe themselves free from any ailment which would disqualify them for service in any climate.

Successful candidates will be numbered according to their attainments on examination, and will be commissioned in the same order as vacancies occur. Upon appointment the young officers are, as a rule, first assigned to duty at one of the large hospitals, as at Boston, New York, New Orleans, Chicago, or San Francisco.

Assistant surgeons receive $1,600, passed assistant surgeons, $2,000, and surgeons, $2,500 a year. The tenure of office is permanent.

For further information, or for invitation to appear before the board of examiners, address "Surgeon-General, Public Health and Marine-Hospital Service, Washington, D. C."

---

The United States Civil Service Commission announces an examination on January 13, 1909, at the usual places, to secure eligibles from which to make certification to fill a vacancy in the position of medical interne (female), Government Hospital for the Insane, Washington, D. C., at $600 per annum with maintenance, and vacancies requiring similar qualifications as they may occur in that hospital. The department states that it reserves the right to terminate the appointment at the expiration of one year of service if it is deemed advisable to do so.

